# Infective Endocarditis in Childhood: a Single-Center Experience of 18 Years

**DOI:** 10.21470/1678-9741-2020-0035

**Published:** 2021

**Authors:** Kahraman Yakut, Zafer Ecevit, Niyazi Kursad Tokel, Birgul Varan, Murat Ozkan

**Affiliations:** 1 Department of Pediatric Infectious Diseases, Baskent University School of Medicine, Ankara, Turkey.; 2 Department of Pediatric Cardiology, Baskent University School of Medicine, Ankara, Turkey.; 3 Department of Cardiovascular Surgery, Baskent University School of Medicine, Ankara, Turkey.

**Keywords:** Endocarditis, *Staphylococcus* Infections, Congenital Heart Defects, Anti-Bacterial Agents, Heart Murmurs

## Abstract

**Introduction:**

We aimed to present the risk factors, clinical and laboratory findings, treatment management, and risk factors for morbidity and mortality of infective endocarditis (IE) as well as to relate experiences at our center.

**Method:**

We retrospectively analyzed data of 47 episodes in 45 patients diagnosed with definite/possible IE according to the modified Duke criteria between May 2000 and March 2018.

**Results:**

The mean age of all patients at the time of diagnosis was 7.6±4.7 years (range: 2.4 months to 16 years). The most common symptoms and findings were fever (89.3%), leukocytosis (80.8%), splenomegaly (70.2%), and a new heart murmur or changing of pre-existing murmur (68%). *Streptococcus viridans* (19.1%), *Staphylococcus aureus* (14.8%), and coagulase-negative *Staphylococci* (10.6%) were the most commonly isolated agents. IE-related complications developed in 27.6% of the patients and the mortality rate was 14.8%.

**Conclusion:**

We found that congenital heart disease remains a significant risk factor for IE. The highest risk groups included operated patients who had conduits in the pulmonary position and unoperated patients with a large ventricular septal defect. Surgical intervention was required in most of the patients. Mortality rate was high, especially in patients infected with *S. aureus*, although the time between the onset of the first symptom and diagnosis was short. Patients with fever and a high risk of IE should be carefully examined for IE, and evaluation in favor of IE until proven otherwise will be more accurate. In high-risk patients with prolonged fever, IE should be considered in the differential diagnosis.

**Table:** 

Abbreviations, acronyms & symbols				
**ABV**	**= Aortic balloon valvuloplasty**		**IVC**	**= Inferior vena cava**
**ALT**	**= Alanine aminotransferase**		**L-AVVR**	**= Left atrioventricular valve regurgitation**
**APV**	**= Absent pulmonary valve**		**LA**	**= Left atrium**
**AR**	**= Aortic regurgitation**		**LVOT**	**= Left ventricle outflow tract**
**ARF**	**= Acute rheumatic fever**		**MR**	**= Mitral regurgitation**
**AS**	**= Aortic stenosis**		**MV**	**= Mitral valve**
**ASD**	**= Atrial septal defect**		**MVR**	**= Mitral valve replacement**
**ASO**	**= Arterial switch operation**		**NICU**	**= Neonatal intensive care unit**
**AST**	**= Aspartate aminotransferase**		**PA**	**= Pulmonary atresia**
**AV**	**= Aortic valve**		**PAVSD**	**= Partial atrioventricular septal defect**
**AVR**	**= Aortic valve replacement**		**PC**	**= Pulmonary conduit**
**BAV**	**= Bicuspid aortic valve**		**PS**	**= Pulmonary stenosis**
**BCA**	**= Balloon coarctation angioplasty**		**PTFE**	**= Polytetrafluoroethylene**
**BT**	**= Blalock-Taussig**		**PV**	**= Pulmonary valve**
**C-TGA**	**= Corrected transposition of the great arteries**		**R-AVV**	**= Right atrioventricular valve**
**CAVSD**	**= Complete atrioventricular septal defect**		**R-AVVR**	**= Right atrioventricular valve regurgitation**
**CHD**	**= Congenital heart disease**		**RA**	**= Right atrium**
**CIED**	**= Cardiac implantable electronic device**		**RHD**	**= Rheumatic heart diseases**
**CoA**	**= Coarctation of the aorta**		**RV-PA**	**= Right ventricular–pulmonary artery**
**CoNS**	**= Coagulase-negative Staphylococci**		**RVOTR**	**= Right ventricular outflow tract reconstruction**
**CRP**	**= C-reactive protein**		**SVC**	**= Superior vena cava**
**CT**	**= Computed tomography**		**TEE**	**= Transesophageal echocardiography**
**DORV**	**= Double outlet right ventricle**		**TGA**	**= Transposition of the great arteries**
**DSM**	**= Discrete subaortic membrane**		**TOF**	**= Tetralogy of Fallot**
**HACEK**	**= Haemophilus species, Aggregatibacter species, Cardiobacterium hominis, Eikenella corrodens, and Kingella species**		**TTE**	**= Transthoracic echocardiography**
			**TV**	**= Tricuspid valve**
**IE**	**= Infective endocarditis**		**VSD**	**= Ventricular septal defect**

## INTRODUCTION

Infective endocarditis (IE), which develops because of bacterial, viral, or fungal agents, is an infection of the endocardium. Although IE often affects the heart valves, it may also cause septal defects or mural endocarditis. IE is less common in children than in adults, and it is associated with high morbidity and mortality due to prolonged treatment time and related complications. The incidence of IE in children is 0.43 per 100,000 children^[1]^. Its risk factors include congenital heart disease (CHD), rheumatic heart diseases (RHD), and bacteremia due to hospital-acquired infections, with CHD being the main risk factor. Currently, increased invasive interventions and long-term use of central venous catheters increase the risk of IE in patients without an underlying heart disease.

In this study, we present the clinical, diagnostic, and microbiological characteristics of patients with IE who were followed up at a tertiary care hospital that is an important pediatric cardiovascular and cardiac surgery center in Turkey.

## METHODS

The data of pediatric patients (aged ≤ 18 years) diagnosed with definite/possible IE episodes according to the modified Duke criteria^[2]^ and followed up at our clinic between May 2000 and March 2018 were retrospectively analyzed. This study was approved by the Ethics Committee of our university (KA17/216-15.08.2017). The clinical and laboratory findings, demographic and echocardiographic characteristics, pathological and culture findings, vegetation localization, underlying disease, acquired heart valve diseases, central catheter or cardiac implantable electronic device (CIED) use, previous surgical interventions, complications, transthoracic echocardiography (TTE) and transesophageal echocardiography (TEE) findings, history of broad-spectrum antibiotic use, history of hospitalization in a long-term intensive care unit, and length of hospital stay were evaluated in all patients. Furthermore, the erythrocyte sedimentation rate, C-reactive protein (CRP) level, complete blood count, and liver and kidney functions were examined before and after treatment. TTE was performed in all patients, and TEE and/or multislice computed tomography (CT) was performed if needed.

CHD, which was included in the risk group, was examined in two subgroups, *i.e.*, simple and complex CHD. Other risk factors included acquired heart disease, malignancy, history of broad-spectrum antibiotic use, history of hospitalization in a long-term intensive care unit, and use of central catheter or CIEDs for another disease.

Patients who met the pathological criteria according to the modified Duke criteria and those who met two major criteria, one major and three minor criteria, or five minor criteria according to the clinical criteria were considered to have definite IE, whereas those who met one major and one minor criterion or three minor criteria were considered to have possible IE. Repeated IE occurrence after the treatment of the first episode was defined as recurrent IE. In cases in which the appearance of endocarditis symptoms or positive blood cultures was observed 48 h after admission or in which IE was diagnosed within eight weeks after cardiac surgery or catheterization, the patients were considered to have nosocomial IE.

### Statistical Analysis

Statistical analysis was performed using the PASW version 17.0 software (SPSS Inc., Chicago, Illinois, United States of America). Descriptive statistics were expressed as mean ± standard deviation and frequency. A P-value < 0.05 was considered statistically significant.

## RESULTS

### Demographic Characteristics

A total of 47 IE episodes were detected in 45 patients. Among these IE episodes, 34 (72.3%) were definite and 13 (27.6%) were possible IE episodes according to the modified Duke criteria. The mean age of the patients at the time of diagnosis was 7.6±4.7 years (range: 2.4 months to 16 years). In addition, 36 IE episodes (76.6%) were noted in males and 11 (23.4%) in females. Eight patients (17%) were aged < 1 year, seven (14.8%) were aged 2-5 years, 20 (42.5%) were aged 6-10 years, and 12 (25.5%) were aged 11-18 years.

At least one risk factor was observed in all IE episodes, with the most common risk factor being CHD (n=41, 87.2%). Furthermore, 22 IE episodes (46.8%) involved complex CHD and 19 (40.4%) involved simple CHD. The most notable CHD included patients who underwent surgery and had conduit insertion at the pulmonary position (29.8%) as well as those with untreated ventricular septal defect (VSD) (23.4%) (Table 1). Risk factors other than CHD included heart valve disease associated with RHD in three patients (6.4%), a history of long-term central catheterization and follow-up after broad-spectrum antibiotic therapy in two patients (4.3%), and a history of permanent dialysis catheter inserted into the right subclavian vein in one patient (2.1%). Three patients had a prosthetic valve; one of these patients underwent aortic valve replacement due to severe aortic insufficiency associated with RHD, one underwent mitral valve replacement due to severe mitral valve insufficiency associated with RHD, and one underwent aortic valve replacement after balloon valvuloplasty due to bicuspid aortic valve disease and aortic valve stenosis. The time between prosthetic valve replacement and IE in these patients was three months, two years, and eight years, respectively. One patient developed complete atrioventricular block after surgery to repair tetralogy of Fallot and had a dual-chamber pacemaker.

**Table 1:** . Diagnosis of patients and surgical procedures before infective endocarditis episodes.

Diagnosis	Surgical	Foreign material	Vegetation area
VSD	No		MV, VSD-left side
VSD	No		VSD-right side
VSD	No		Native-PV
VSD	No		TV, PV
VSD	No		MV
VSD	No		TV
VSD	No		TV
VSD	No		TV
VSD	No		TV, PV
VSD	No		Abscess between the RA and aorta
VSD	No		VSD-right side
VSD	VSD-repair		TV
PAVSD, MR, TR, DSM	PAVSD-repair		AV
TOF	TOF-complete repair		VSD patch-right side
TOF	Modified BT shunt	PTFE graft	TV
TOF	TOF-complete repair	Biological valved conduit	PC
TOF, atrioventricular block	TOF-complete repair	Biological valved conduit, dual chamber pacemaker lead	PC
BAV, AS	Modified Konno, AVR, MVR	Prosthetic MV and AV	Prosthetic MV
BAV, AS, MR	ABV		AV
BAV, AS, AR	Ross	Biological valved conduit	AV
BAV, AS, MR	ABV		AV
CoA, DSM	BCA		MV
DSM, AR	DSM resection		LVOT, AV
TGA, ASD	ASO, ASD-repair		LVOT, AV
TGA, ASD	ASO, ASD-repair		MV, LA
TGA, VSD, ASD, PS (mild)	ASO, VSD and ASD-repair		Absent vegetation
TGA, VSD, ASD, PS (mild)	ASO, VSD and ASD-repair		AV, PV
TGA, VSD, ASD, PS	Rastelli surg, ASD-repair	Biological valved conduit	PC
TGA, VSD, ASD, PS	Rastelli surg, ASD-repair	Biological valved conduit	PC
TGA, VSD, ASD, PS	Modified BT shunt	PTFE graft	Native-PV
DORV, VSD, PS	Rastelli surg, RVOTR with conduit	Biological valved conduit	PC
DORV, VSD, PS	Rastelli surg, RVOTR with conduit	Biological valved conduit	PC
DORV, VSD, PS	Rastelli surg, RVOTR with conduit	Biological valved conduit	TV
DORV, VSD	Pulmonary banding		Absent vegetation
APV, VSD	RVOTR with conduit, VSD repair	Biological valved conduit	PC
APV, VSD	RVOTR with conduit, VSD repair	Biological valved conduit	PC
PA, VSD	RVOTR with conduit, VSD repair	Biological valved conduit	PC
PA, VSD	RVOTR with conduit, VSD repair	Biological valved conduit	PC
Truncus arteriosus Type 1	RVOTR with conduit, VSD repair	Biological valved conduit	PC
C-TGA, VSD, PS, dextrocardia	RVOTR with conduit, VSD repair	Biological valved conduit	PC
CAVSD, L-AVVR, R-AVVR	No		R-AVV
Chronic kidney failure	No	Dialysis catheter	TV
Premature-NICU hosp	No	Central catheter	RA fungus ball
Chronic malnutrition	No	Central catheter	IVC-RA fungus ball
ARF, AR, MR	No		AV
ARF, MR, AR	MVR	Prosthetic MV	LA
ARF, AR	AVR	Prosthetic AV	Prosthetic AV

ABV=aortic balloon valvuloplasty; APV=absent pulmonary valve; AR=aortic regurgitation; ARF=acute rheumatic fever; AS=aortic stenosis; ASD=atrial septal defect; ASO=arterial switch operation; AV=aortic valve; AVR=aortic valve replacement; BAV=bicuspid aortic valve; BCA=balloon coarctation angioplasty; BT=Blalock-Taussig; C-TGA=corrected transposition of the great arteries; CAVSD=complete atrioventricular septal defect; CoA=coarctation of the aorta; DORV=double outlet right ventricle; DSM=discrete subaortic membrane; IVC=inferior vena cava; L-AVVR=left atrioventricular valve regurgitation; LA=left atrium; LVOT=left ventricle outflow tract; MR=mitral regurgitation; MV=mitral valve; MVR=mitral valve replacement; NICU=neonatal intensive care unit; PA=pulmonary atresia; PAVSD=partial atrioventricular septal defect; PC=pulmonary conduit; PS=pulmonary stenosis; PTFE=polytetrafluoroethylene; PV=pulmonary valve; R-AVV=right atrioventricular valve; R-AVVR=right atrioventricular valve regurgitation; RA=right atrium; RVOTR=right ventricular outflow tract reconstruction; TGA=transposition of the great arteries; TOF=tetralogy of Fallot; TV=tricuspid valve; TR=Tricuspid regurgitation; VSD=ventricular septal defect

Notably, 25.5% of the patients had a history of infection in the month before IE. Of these, three patients had pneumonia, two had acute gastroenteritis, one had open wound infection, and six had untreated dental caries. Furthermore, 59.6% of the patients had undergone open heart surgery for CHD or acquired heart disease and 6.4% received a therapeutic intervention before the IE episodes. In our study, 10% of the IE episodes developed in the initial six months after cardiac surgery. The mean time between previous surgery and/or therapeutic intervention and IE attack was 4.5±3.7 years (minimum: 3.6 months; maximum: 13 years). Moreover, 34% of the patients had no history of interventional cardiac procedures or cardiac surgery before IE.

IE episodes were community-acquired in 44 patients (93.6%), and IE was associated with intracardiac foreign bodies in five patients. Three patients had hospital-acquired IE. Two of the latter patients were followed up after central catheterization with broad-spectrum antibiotic therapy in the intensive care unit, but echocardiographic examination (performed because of reproduction of *Candida albicans* in repeated blood cultures) revealed a fungus ball.

Fourteen IE episodes occurred in patients with a biological valved conduit in the pulmonary position. All were bovine jugular vein conduits (Contegra^TM^ Pulmonary Valved Conduit, Medtronic, Minneapolis, United States of America). None of the patients had received a homograft in the pulmonary position.

In 55.3% of the patients, diagnosis could be performed in the initial 10 days after the onset of symptoms. The mean time from the onset of the first symptom to diagnosis was 10.9 days (range: 4-32 days). An increase in the incidence of IE has been observed after 2010, with 31 patients (66%) being diagnosed between 2011 and 2017.

### Clinical Finding

The most common symptoms and signs detected in the patients were fever (≥ 38 °C) (89.3%), leukocytosis (80.8%), splenomegaly (70.2%), new heart murmur or changing of pre-existing murmur (68%), fatigue (61.7%), and anorexia (55.3%). The mean erythrocyte sedimentation rate was 45.6 mm/h (range: 8-86 mm/h), and the mean CRP was 74.6 mg/dl (range: 10-130 mg/dl) (Table 2). Congestive heart failure was noted in 14.9% of the patients at the time of diagnosis. Accompanying immunological and vascular phenomena included septic pulmonary embolism in 14.9% of the IE episodes, rheumatoid factor positivity in 12.7%, pulmonary embolism in 10.6%, Janeway lesions in 10.6%, splinter hemorrhages in 8.5%, and major arterial embolism in 8.5%.

**Table 2:** Clinical and laboratory features of patients with infective endocarditis episode.

Endocarditis episode (n=47)	Mean (min-max)	n (%)
Female/male		nov/34
Age of patients (years)	7.6 years (72 days-16 years)	
Fever (≥ 38ºC)		42 (89.3)
Splenomegaly		33 (70.2)
New murmur/change of murmur		32 (68)
Body weight loss in patients		18 (38.2)
Hepatomegaly		16 (34)
Hematuria		10 (21.2)
Arthritis/arthralgia		4 (8.5)
Leukocytosis		38 (80.8)
Erythrocyte sedimentation velocity (mm/h)	45.6 (8-86)	
C-reactive protein (mg/dl)	74.6 (10-130)	
Anemia	According to age	18 (38.3)
Tachycardia	According to age	22 (46.8)
Cough		20 (42.5)
Debility		29 (61.7)
Myalgia		10 (21.3)
Abdominal pain		3 (6.4)
Anorexia		26 (55.3)
Dyspnea		14 (29.8)
Chest pain		8 (17)
Rheumatoid factor positivity		6 (12.7)
Janeway lesion		5 (10.6)
Glomerulonephritis		3 (6.4)
Osler nodule		2 (4.2)
Roth spot		3 (6.4)
Splinter hemorrhage		4 (8.5)
AST elevation		2 (4.2)
ALT elevation		5 (10.6)
Creatinine elevation		1 (2.1)
Headache		2 (4.2)
Vomiting		2 (4.2)

ALT=alanine aminotransferase; AST=aspartate aminotransferase

### Echocardiography

**Fig. 1:**
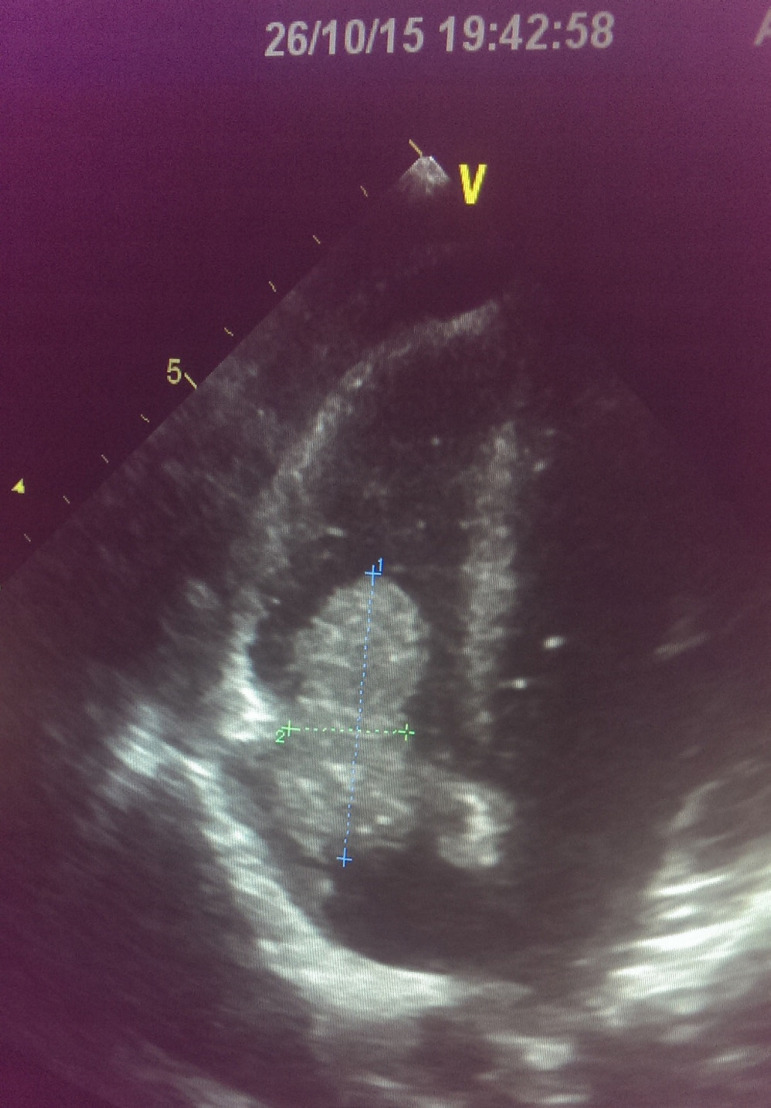
In a patient with a permanent dialysis catheter, vegetation is observed on the tricuspid valve.

Vegetation was observed via TTE in 40 patients (85.1%) with IE episodes (Figure 1) and via TEE in five of seven without vegetation observed on TTE. These seven patients showed growth of the causative microorganism in their blood cultures. Two patients who did not show vegetation on TTE and TEE were treated as patients with IE because they showed microorganism growth in repeated blood cultures and IE diagnosis was supported based on meeting the minor criteria. In IE, vegetation was observed in the right heart structures in 62.2% of the patients, in the left heart structures in 31.1%, and in structures of both sides in 4.4% (Table 3).

**Table 3:** Vegetation location and frequency.

Vegetation location	n (%)
Pulmonary valve	16 (30.7)
Tricuspid valve	10 (19.2)
Aortic valve	10 (19.2)
Mitral valve	5 (9.6)
Right ventricle	3 (5.7)
Right atrium	3 (5.7)
Left ventricle	3 (5.7)
Left atrium	1 (1.9)
Inferior vena cava	1 (1.9)
Total	52 (100)

Note: Some patients had vegetation in multiple areas.

### Causative Microorganisms

In 68.1% of the IE episodes (32 patients), growth of the causative microorganism in blood culture was observed. *Streptococcus viridans* (19.1%), *Staphylococcus aureus* (14.8%), and coagulase-negative *Staphylococci* (CoNS) (10.6%) were the most commonly isolated agents (Table 4). Central catheterization was associated with IE in one of five patients with coagulase-negative staphylococcal endocarditis. In 31.9% of the IE episodes, no microorganism growth in blood culture was observed. Microorganisms from the *Haemophilus* species, *Aggregatibacter* species, Cardiobacterium *hominis, Eikenella corrodens*, and *Kingella* species (HACEK) group were detected in two patients. *Eikenella corrodens* reproduction was detected in one patient and *Cardiobacterium hominis* reproduction in the other. *C. albicans*-induced fungus balls were detected in two patients who were followed up after central catheterization with broad-spectrum antibiotic therapy. Furthermore, 60% of patients with culture-negative IE received antibiotic treatment before the blood culture test.

**Table 4:** Microorganisms isolated in infective endocarditis episode.

Microorganisms	Endocarditis episode (n)	Incidence (%)
*Streptococcus viridans*	9	19.1
*Staphylococcus aureus*	7	14.8
Methicillin-resistant	4	
Methicillin-sensitive	3	
*Coagulase-negative staphylococci*	5	10.6
Methicillin-resistant	3	
Methicillin-sensitive	2	
*Eikenella corrodens*	1	2.1
*Cardiobacterium hominis*	1	2.1
*Pseudomonas aeruginosa*	2	4.2
*Candida albicans*	2	4.2
*Enterococcus fecalis*	2	4.2
*Brucella melitensis*	1	2.1
*Escherichia coli*	1	2.1
*Burkholderia cepacia*	1	2.1
*Culture negative endocarditis*	15	31.9
**Total**	**47**	**100**

### Prognosis

In two patients, IE recurred within a six-month interval. Of these, one patient underwent arterial switch operation following the diagnosis of transposition of the great arteries (TGA), VSD, and atrial septal defect (ASD). In blood cultures, CoNS and *Enterococcus faecalis* growth was noted. One patient who had undergone total surgical correction using conduit had a double-outlet right ventricle, VSD, and pulmonary stenosis (PS). This patient underwent a second conduit replacement due to conduit vegetation. In blood cultures, CoNS and *S. aureus* growth was noted.

Congestive heart failure developed in one patient while receiving antibiotic treatment following the diagnosis of VSD and pneumonia. Echocardiographic examination of this patient revealed a 20-×25-mm abscess between the right atrium and aorta. In blood culture, vancomycin-resistant *S. aureus* growth was noted. In this patient, while receiving antibiotic and anticongestive therapies, septic shock developed within a short period and the patient died despite all interventions. All patients were treated with antibiotic treatment. In addition to antibiotic treatment, 37 of the IE episodes (78.7%) were treated with surgical intervention. The mean duration of preoperative antibiotic use was 8.5±2.2 days (range: 5-12 days). Surgical indications were determined to be the removal of infected prosthetic material in 14 patients, intervention for CHD accompanying IE in 11, removal of large and mobile vegetation in seven, severe valve dysfunction in four, and removal of fungus ball in two.

IE-related complications developed in 27.6% of the patients (Table 5). Left hemiparesis due to intracranial embolism developed in two patients and total occlusion in the right superior vena cava in one patient. The mean length of hospital stay of all patients was 44.6±9.7 days (range: 18-65 days). Additionally, 77.7% of the patients recovered without any sequelae with antibiotic and/or surgical treatment, but 14.8% of the IE episodes resulted in death. *S. aureus* reproduced in three of seven cases that resulted in death.

**Table 5:** Complications in patients with infective endocarditis episode.

Patients (n=13)	n (27.6%)
Heart failure	7 (14.9%)
Septic pulmonary infarction	7 (14.9%)
Pulmonary embolism	5 (10.6%)
Pneumonia	5 (10.6%)
Pleural effusion	4 (8.5%)
Major arterial embolism	4 (8.5%)
Intracranial embolism	3 (6.4%)
Pericardial effusion	3 (6.4%)
Left hemiparesis	2 (4.2%)
Hemoptysis	1 (2.1%)
Mycotic aneurysm	1 (2.1%)
Convulsion	1 (2.1%)
Paresthesia in the left leg	1 (2.1%)
Altered mental status	1 (2.1%)
SVC occlusion	1 (2.1%)
Tricuspid valve injury	1 (2.1%)

Note: Some patients have multiple complications. SVC=superior vena cava

## DISCUSSION

IE is more common in adults than in children. However, as a result of the increase in invasive surgery and medical interventions, particularly for cardiovascular diseases, the incidence of IE in children has started to increase^[3]^. Because of a decrease in the incidence of RHD-associated heart valve diseases in children in developed countries, IE usually develops secondary to CHD^[3-5]^. However, in these countries, while there has been a decrease in the number of patients with underlying RHD-associated heart valve disease, there has been a significant increase in the incidence of prosthetic valve endocarditis^[6,7]^. Reportedly, IE involvement in native pulmonary valve is very rare^[8,9]^. In our study, the underlying risk factor for IE was CHD in 85% of the IE episodes. RHD was a risk factor in a small proportion of our patients (6.4%), possibly because our center is a reference center for CHD. In the literature, pediatric patients diagnosed with IE are accompanied by CHD with a range of 41.7%-81%^[4,5,10-14]^.

In many studies, the risk of IE in CHDs was found to be higher in the left heart structures^[15-17]^. In contrast to findings reported in the literature, vegetation in IE was determined mostly in the right heart structures in most of our patients (62.2%).

In epidemiological studies, the incidence of IE in the general population has been calculated to be 15-60/1,000,000 individuals per year in adults and 3.9-6.4/1.000.000 individuals-year in children^[18,19]^. While the incidence of IE is 11/10,000 individuals-year in adults with CHD, this rate was reported to be three times lower (4.1/10,000 individuals-year) in the pediatric group^[15,17]^. In a study by Rosenthal et al.^[3]^, the predisposing factor was identified as heart disease in 72% of patients with IE. In the literature, the incidence of IE in CHD has been reported to be 35%-51% in children with complex, cyanotic CHD^[5,12,13]^. In our study, the incidence of CHD was as high as 85% probably because children with CHD are monitored more intensively at our center.

Although a gradual improvement in surgical treatments for CHD has led to prolonged survival in these patients, it has also increased the risk of long-term complications such as IE. Rushani et al.^[15]^ reported that children with CHD aged < 3 years had a relatively high risk of IE within six months after cardiac surgery, and this risk has increased by five times in these patients. Approximately 60% of our patients had a history of cardiac surgery before IE, with 10% of the IE episodes developed in the initial six months after cardiac surgery.

In recent years, with the improvements in surgical techniques, the survival rate in patients with CHD has increased. Increase in IE incidence has been observed with intracardiac prosthetic valve replacement, pacemaker implantation, and increased endovascular treatment^[20,21]^. The prosthetic material not only provides a surface that is susceptible to bacterial colonization but it is also often difficult to sterilize^[15]^. Complications such as abscess formation are considered to be more common in cases of prosthetic valve IE, but perivalvular abscess was not observed in the biological valved conduit or prosthetic valve cases in our study. In a study by Khoo et al.^[14]^ involving 138 IE cases, 19.6% of patients had previously undergone a heart valve replacement. In our study, biological valved conduit in right ventricular-pulmonary artery (RV-PA) position in 14 patients, prosthetic aortic valve in two patients, and prosthetic mitral valve in one patient were important risk factors for IE.

In a study, Day et al.^[11]^ reported that the incidence of IE showed a bimodal age distribution wherein it was most frequently observed in infants aged between 31 days and 11 months and in individuals aged 17-20 years. Gupta et al.^[1]^ and Johnson et al.^[4]^ found that most patients diagnosed with IE were in the 6-18-year age group. Similarly, in our study, 68% of the patients were in the 6-18-year age group.

Although Beth Israel and other diagnostic criteria have been used for the diagnosis of IE in the past, the modified Duke criteria, which have higher sensitivity and specificity, are currently used in IE diagnosis^[22,23]^. Their use results in a higher definitive diagnosis rate (range: 70.8%-88%)^[10,22-24]^. On the other hand, in the presence of prosthetic valves or intracardiac devices, the sensitivity of the modified Duke criteria is low^[25]^. In our study, the number of patients with a definite diagnosis accounted for 72.3% of all patients.

The most common symptoms of IE are fever and weight loss^[13]^. In our study, fever was the most common symptom followed by weight loss. Because of the early admission of pediatric patients, conditions such as Osler’s nodes and splinter hemorrhages are less common in children than in adults^[13]^. Similarly, the incidence of these conditions was low in our patients. In a study by Martin et al.^[13]^, splenomegaly was detected in 7% of patients, whereas its rate was very high in our patients (68.9%). In IE cases, vascular and immunological phenomena continue to be common (10,26). Issa et al.^[27]^ reported that the time from the onset of symptoms to IE diagnosis was 49.6±64.5 days (range: 1-731 days) and that 25% of cases were diagnosed within the initial 10 days after the onset of symptoms. In our study, 55.3% of the patients were diagnosed with the conditions 10 days after the onset of symptoms. The fact that vascular and immunological phenomena were lower than those reported in the literature may be due to the early diagnosis and early treatment of IE.

TTE and TEE are the most important methods of diagnosis, treatment, and follow-up of patients with IE^[14,20,28,29]^. In various studies, its sensitivity in IE diagnosis was found to be between 51% and 70%, respectively^[4,5,13,30,31]^. Penk et al.^[32]^ reported that TTE had a sensitivity of 97% in pediatric patients with IE weighing < 60 kg. Although the sensitivity of echocardiography has increased in IE diagnosis in recent years, echocardiography positivity has been found to be higher in patients with normal cardiac anatomy and lower in those with complex CHD. Further, it has been considered that high echocardiography positivity in patients with normal cardiac anatomy might be associated with the initial consideration of IE in their differential diagnosis^[5,12,29]^. TEE is reportedly not necessary for IE diagnosis in most of patients because TTE is sufficient for IE diagnosis in childhood^[33]^. In the presence of a prosthetic valve, if there are very small vegetations (< 2 mm), no vegetation, or vegetation has been embolized, identification of vegetation can be extremely difficult even if TEE is used^[34]^. However, compared with TEE, multislice CT has been reported to provide good results in the evaluation of the perivalvular dimension of IE-related valve abnormalities, particularly abscess or pseudoaneurysms^[35]^. In our cases, typical vegetation was seen to a large extend with TTE (85%). In a small number of cases wherein TTE could not determine typical vegetation, TEE was used successfully. When used together, the positivity rate of TTE/TEE was 95.7%. Multislice CT was performed in two patients with a prosthetic valve to confirm the diagnosis.

Webb et al.^[10]^ reported that the left heart structures were involved in 53% of the IE episodes, the right heart structures in 29%, and both structures in 3.5%. They reported that they could not determine the involved heart structures in 14% of the IE episodes. Tseng et al.^[36]^ reported that the right heart structures were involved in 61.2% of IE episodes, left heart structures in 29.3%, and both structures in 6.9%. In our cases, vegetation was determined in all heart structures, although it was more common in the right heart structures (Table 2). In two patients, no vegetation was identified on TTE/TEE.

In IE episodes in childhood, the most common causative agent is *S. viridians*, followed by *S. aureus* and *Enterococcus* spp.^[3-6,11,13,21,24,32,37]^. While the risk of IE caused by *S. aureus*-caused bacteremia was found to be low in previous studies, *S. aureus* has started to be seen more frequently in community- and hospital-acquired IE in the past two decades^[3,10,38-40]^. In our study, the most common microorganisms were *S. viridans, S. aureus*, and CoNS (Table 3). *E. faecalis*-induced IE has been reported at a ratio of 2%-6% in other studies^[3-5,13]^ and 4.2% in our study. *Brucella melitensis*, a gram-negative coccobacillus, was isolated in one patient. Brucellosis (zoonosis transmitted from sheep and goats) is rarely (0.5%) a cause of IE in children^[41]^.

In the HACEK group of microorganisms, which are slow-growing gram-negative bacteria, the most common cause of endocarditis is *H. parainfluenzae*^[42]^. Endocarditis caused by *E. corrodens*, which frequently colonizes the oropharynx, has been reported in only two (3%) of 76 IE episodes by Martin et al.^[13]^ to date. In our study, *E. corrodens* was isolated in one IE episode (2.1%). In the literature, apart from these, no case of IE in children caused by *E. corrodens* has been reported. In a study in Spain, the HACEK group of microorganisms was found to be responsible for only 1.3% of 1209 IE cases in adults, but *E. corrodens* was not isolated from any patient in that study^[43]^.

*Burkholderia cepacia*, which causes infections in patients with cystic fibrosis and granulomatous disease, is a rare cause of IE. In our study, *B. cepacia* was detected as a causative agent in one patient. In this patient, modified Blalock-Taussig shunt was performed at the age of three months following the diagnosis of TGA, VSD, ASD, and PS. Native pulmonary valve IE was observed three months after cardiac surgery, and *B. cepacia* growth was observed in blood cultures. After two-week antibiotic treatment, the vegetations on the pulmonary valve were cleared, RV-PA connection was achieved with conduit insertion, and the Rastelli procedure was performed. To date, *B. cepacia* has been reported as an IE agent only in one newborn^[44]^.

IE-related mortality in the pediatric group is between 4% and 24%^[3-5,11-14,21]^. The main factors affecting the prognosis in IE are the presence and severity of complications, characteristics of the causative microorganism, and echocardiographic findings. High risks of morbidity and mortality have been reported in patients with congestive heart failure, perianal complications, or *S. aureus*-caused endocarditis^[6,10,21]^. In these patients, the need for surgical treatment in the active stage of the disease is high^[6,10,45]^. The most significant risk factors for mortality in patients with no previous heart disease are the neonatal period and *S. aureus*-caused endocarditis, which depend more on frequent cardiac complications in these patients^[5,12,13]^. In our study, no specific distribution in terms of CHD in patients with mortality was observed.

The mortality rate in *Candida*-caused IE has been reported to be approximately 90%^[28]^. In our study, excision of a fungus ball was surgically performed with antifungal treatment in two patients with *Candida* IE, following which full recovery was achieved.

The most common complications in our patients were congestive heart failure, septic pulmonary embolism, pulmonary embolism, and major arterial embolism. Congestive heart failure and embolic phenomena were the most significant factors to have an impact on mortality and morbidity in our patients. In addition, the characteristics of causative microorganisms were associated with poor results. *S. aureus* reproduction was found to be associated with a high rate of mortality in our study. Rapid identification of patients at high risk of death may provide an opportunity to improve the prognosis of IE.

### Limitations

A retrospective study is a significant disadvantage in the collection and evaluation of data.

**Table:** 

Authors' roles & responsibilities	
KY	Substantial contributions to the conception or design of the work; or the acquisition, analysis, or interpretation of data for the work; drafting the work or revising it critically for important intellectual content; agreement to be accountable for all aspects of the work in ensuring that questions related to the accuracy or integrity of any part of the work are appropriately investigated and resolved; final approval of the version to be published
ZE	Substantial contributions to the conception or design of the work; or the acquisition, analysis, or interpretation of data for the work; drafting the work or revising it critically for important intellectual content; agreement to be accountable for all aspects of the work in ensuring that questions related to the accuracy or integrity of any part of the work are appropriately investigated and resolved; final approval of the version to be published
NKT	Substantial contributions to the conception or design of the work; or the acquisition, analysis, or interpretation of data for the work; drafting the work or revising it critically for important intellectual content; agreement to be accountable for all aspects of the work in ensuring that questions related to the accuracy or integrity of any part of the work are appropriately investigated and resolved; final approval of the version to be published
BV	Drafting the work or revising it critically for important intellectual content; agreement to be accountable for all aspects of the work in ensuring that questions related to the accuracy or integrity of any part of the work are appropriately investigated and resolved; final approval of the version to be published
MO	Drafting the work or revising it critically for important intellectual content; agreement to be accountable for all aspects of the work in ensuring that questions related to the accuracy or integrity of any part of the work are appropriately investigated and resolved; final approval of the version to be published

## CONCLUSION

CHD is an increasingly significant risk factor for IE. The presence of a biological valved conduit and unoperated large VSD were two important risk factors for IE in our patients. Furthermore, we observed high mortality rate associated with *S. aureus* infection. Even if echocardiographic examination findings are normal in patients with CHD on follow-up, particularly in those with fever of unknown cause, who have undergone surgery and have been implanted with prosthetic devices, examination and treatment should be performed in favor of IE.

## References

[1] Gupta S, Sakhuja A, McGrath E, Asmar B (2017). Trends, microbiology, and outcomes of infective endocarditis in children during 2000-2010 in the United States. Congenit Heart Dis.

[2] Li JS, Sexton DJ, Mick N, Nettles R, Fowler VG Jr, Ryan T (2000). Proposed modifications to the Duke criteria for the diagnosis of infective endocarditis. Clin Infect Dis.

[3] Rosenthal LB, Feja KN, Levasseur SM, Alba LR, Gersony W, Saiman L (2010). The changing epidemiology of pediatric endocarditis at a children’s hospital over seven decades. Pediatr Cardiol.

[4] Johnson JA, Boyce TG, Cetta F, Steckelberg JM, Johnson JN (2012). Infective endocarditis in the pediatric patient: a 60-year single-institution review. Mayo Clin Proc.

[5] Coward K, Tucker N, Darville T (2003). Infective endocarditis in Arkansan children from 1990 through 2002. Pediatr Infect Dis J.

[6] Ware AL, Tani LY, Weng HY, Wilkes J, Menon SC (2014). Resource utilization and outcomes of infective endocarditis in children. J Pediatr.

[7] Tleyjeh IM, Abdel-Latif A, Rahbi H, Scott CG, Bailey KR, Steckelberg JM (2007). A systematic review of population-based studies of infective endocarditis. Chest.

[8] Liekiene D, Bezuska L, Semeniene P, Cypiene R, Lebetkevicius V, Tarutis V (2019). Surgical treatment of infective endocarditis in pulmonary position-15 years single centre experience. Medicina (Kaunas).

[9] Deng H, Ma Y, Zhai H, Miao Q (2013). Surgical valve repair of isolated pulmonary valve endocarditis. Interact Cardiovasc Thorac Surg.

[10] Webb R, Voss L, Roberts S, Hornung T, Rumball E, Lennon D (2014). Infective endocarditis in New Zealand children 1994-2012. Pediatr Infect Dis J.

[11] Day MD, Gauvreau K, Shulman S, Newburger JW (2009). Characteristics of children hospitalized with infective endocarditis. Circulation.

[12] Saiman L, Prince A, Gersony WM (1993). Pediatric infective endocarditis in the modern era. J Pediatr.

[13] Martin JM, Neches WH, Wald ER (1997). Infective endocarditis: 35 years of experience at a children’s hospital. Clin Infect Dis.

[14] Khoo B, Buratto E, Fricke TA, Gelbart B, Brizard CP, Brink J (2019). Outcomes of surgery for infective endocarditis in children: a 30-year experience. J Thorac Cardiovasc Surg.

[15] Rushani D, Kaufman JS, Ionescu-Ittu R, Mackie AS, Pilote L, Therrien J (2013). Infective endocarditis in children with congenital heart disease: cumulative incidence and predictors. Circulation.

[16] Wilson W, Taubert KA, Gewitz M, Lockhart PB, Baddour LM, Levison M (2007). Prevention of infective endocarditis: guidelines from the American heart association: a guideline from the American heart association rheumatic fever, endocarditis, and Kawasaki disease committee, council on cardiovascular disease in the young, and the council on clinical cardiology, council on cardiovascular surgery and anesthesia, and the quality of care and outcomes research interdisciplinary working group. Circulation.

[17] Verheugt CL, Uiterwaal CS, van der Velde ET, Meijboom FJ, Pieper PG, Veen G (2011). Turning 18 with congenital heart disease: prediction of infective endocarditis based on a large population. Eur Heart J.

[18] Iung B, Vahanian A (2011). Epidemiology of valvular heart disease in the adult. Nat Rev Cardiol.

[19] Schollin J, Bjarke B, Wesström G (1986). Infective endocarditis in Swedish children. II. Location, major complications, laboratory findings, delay of treatment, treatment and outcome. Acta Paediatr Scand.

[20] Yuan XC, Liu M, Hu J, Zeng X, Zhou AY, Chen L (2019). Diagnosis of infective endocarditis using echocardiography. Medicine (Baltimore).

[21] Cahill TJ, Jewell PD, Denne L, Franklin RC, Frigiola A, Orchard E (2019). Contemporary epidemiology of infective endocarditis in patients with congenital heart disease: a UK prospective study. Am Heart J.

[22] Stockheim JA, Chadwick EG, Kessler S, Amer M, Abdel-Haq N, Dajani AS (1998). Are the Duke criteria superior to the Beth Israel criteria for the diagnosis of infective endocarditis in children?. Clin Infect Dis.

[23] Tissières P, Gervaix A, Beghetti M, Jaeggi ET (2003). Value and limitations of the von Reyn, Duke, and modified Duke criteria for the diagnosis of infective endocarditis in children. Pediatrics.

[24] Lin YT, Hsieh KS, Chen YS, Huang IF, Cheng MF (2013). Infective endocarditis in children without underlying heart disease. J Microbiol Immunol Infect.

[25] Habib G, Lancellotti P, Antunes MJ, Bongiorni MG, Casalta JP, Del Zotti F (2015). 2015 ESC guidelines for the management of infective endocarditis: the task force for the management of infective endocarditis of the European society of cardiology (ESC). Endorsed by: European association for cardio-thoracic surgery (EACTS), the European association of nuclear medicine (EANM). Eur Heart J.

[26] Thuny F, Di Salvo G, Belliard O, Avierinos JF, Pergola V, Rosenberg V (2005). Risk of embolism and death in infective endocarditis: prognostic value of echocardiography: a prospective multicenter study. Circulation.

[27] Issa VS, Fabri J Jr, Pomerantzeff PM, Grinberg M, Pereira-Barreto AC, Mansur AJ (2003). Duration of symptoms in patients with infective endocarditis. Int J Cardiol.

[28] Habib G, Hoen B, Tornos P, Thuny F, Prendergast B, Vilacosta I (2009). Guidelines on the prevention, diagnosis, and treatment of infective endocarditis (new version 2009): the task force on the prevention, diagnosis, and treatment of infective endocarditis of the European society of cardiology (ESC). Endorsed by the European society of clinical microbiology and infectious diseases (ESCMID) and the International society of chemotherapy (ISC) for infection and cancer. Eur Heart J.

[29] Kelly P, Hua N, Madriago EJ, Holmes KW, Shaughnessy R, Ronai C (2020). The utility of echocardiography in pediatric patients with structurally normal hearts and suspected endocarditis. Pediatr Cardiol.

[30] Awadallah SM, Kavey RE, Byrum CJ, Smith FC, Kveselis DA, Blackman MS (1991). The changing pattern of infective endocarditis in childhood. Am J Cardiol.

[31] Fukushige J, Igarashi H, Ueda K (1994). Spectrum of infective endocarditis during infancy and childhood: 20-year review. Pediatr Cardiol.

[32] Penk JS, Webb CL, Shulman ST, Anderson EJ (2011). Echocardiography in pediatric infective endocarditis. Pediatr Infect Dis J.

[33] Humpl T, McCrindle BW, Smallhorn JF (2003). The relative roles of transthoracic compared with transesophageal echocardiography in children with suspected infective endocarditis. J Am Coll Cardiol.

[34] Hill EE, Herijgers P, Claus P, Vanderschueren S, Peetermans WE, Herregods MC (2007). Abscess in infective endocarditis: the value of transesophageal echocardiography and outcome: a 5-year study. Am Heart J.

[35] Feuchtner GM, Stolzmann P, Dichtl W, Schertler T, Bonatti J, Scheffel H (2009). Multislice computed tomography in infective endocarditis: comparison with transesophageal echocardiography and intraoperative findings. J Am Coll Cardiol.

[36] Tseng WC, Chiu SN, Shao PL, Wang JK, Chen CA, Lin MT (2014). Changing spectrum of infective endocarditis in children: a 30 years experiences from a tertiary care center in Taiwan. Pediatr Infect Dis J.

[37] Murdoch DR, Corey GR, Hoen B, Miró JM, Fowler VG Jr, Bayer AS (2009). Clinical presentation, etiology, and outcome of infective endocarditis in the 21st century: the international collaboration on endocarditis-prospective cohort study. Arch Intern Med.

[38] Pasquali SK, He X, Mohamad Z, McCrindle BW, Newburger JW, Li JS (2012). Trends in endocarditis hospitalizations at US children’s hospitals: impact of the 2007 American heart association antibiotic prophylaxis guidelines. Am Heart J.

[39] Mylotte JM, McDermott C, Spooner JA (1987). Prospective study of 114 consecutive episodes of Staphylococcus aureus bacteremia. Rev Infect Dis.

[40] Millar BC, Prendergast BD, Moore JE (2008). Community-associated MRSA (CA-MRSA): an emerging pathogen in infective endocarditis. J Antimicrob Chemother.

[41] Kazaz H, Celkan MA, Ustunsoy H, Baspinar O (2005). Mitral annuloplasty with biodegradable ring for infective endocarditis: a new tool for the surgeon for valve repair in childhood. Interact Cardiovasc Thorac Surg.

[42] Feder HM Jr, Roberts JC, Salazar J, Leopold HB, Toro-Salazar O (2003). HACEK endocarditis in infants and children: two cases and a literature review. Pediatr Infect Dis J.

[43] Ambrosioni J, Martinez-Garcia C, Llopis J, Garcia-de-la-Maria C, Hernández-Meneses M, Tellez A (2018). HACEK infective endocarditis: epidemiology, clinical features, and outcome: a case-control study. Int J Infect Dis.

[44] Yonas E, Damay V, Pranata R, Nusarintowati N (2018). Infective endocarditis due to Burkholderia cepacia in a neonate: a case report. J Med Case Rep.

[45] Rovery C, Greub G, Lepidi H, Casalta JP, Habib G, Collart F (2005). PCR detection of bacteria on cardiac valves of patients with treated bacterial endocarditis. J Clin Microbiol.

